# Cell death as a defense strategy against pathogens in plants and animals

**DOI:** 10.1371/journal.ppat.1011253

**Published:** 2023-04-06

**Authors:** Jose Salguero-Linares, Nuria S. Coll

**Affiliations:** 1 Centre for Research in Agricultural Genomics (CRAG), CSIC-IRTA-UAB-UB, Campus UAB, Bellaterra, Barcelona, Spain; 2 Consejo Superior de Investigaciones Científicas (CSIC), Barcelona, Spain; Shanghai Center for Plant Stress Biology, CHINA

## Abstract

Eukaryotes are endowed with sophisticated innate immune systems to recognize non-self and halt pathogen proliferation. Activation of cell death at the site of attempted pathogen ingress is a common strategy used by plants and animals to restrict pathogen proliferation and trigger immune responses in the surrounding tissues. As such, immunogenic cell death shares several features in both plants and animals that will be discussed in this article, namely: (i) it is triggered by activation of NLR immune receptors—often through oligomerization; (ii) it results in disruption of the plasma membrane (PM)/endomembrane integrity driving an imbalance in ion fluxes; and (iii) it results in the release of signaling molecules from dying cells.

## 1. Pathogens are perceived by immune receptors

Immune receptors of the nucleotide-binding leucine-rich repeat (NLR)-type constitute fundamental elements of the plant and animal innate immune systems (Table 1). Animal NLRs respond to and mediate interaction with pathogen- or danger-associated molecular patterns (PAMPs or DAMPs) [1]. In plants, the task of pathogen recognition is divided between intracellular NLRs and cell surface pattern-recognition receptors (PRRs). While plant NLRs recognize secreted pathogen effectors or their activity within the host cells, PRRs recognize PAMPs [2]. Animal and plant NLRs share a similar multidomain architecture within the core nucleotide-binding and oligomerization domain (NOD) and the leucine-rich repeat (LRR) domains. However, there is substantial diversity at the C- and N-terminal accessory domains [3].

**Table 1:** Abbreviation list.

Abbreviation	Description of term
ASC	Apoptosis-associated speck-like protein containing a caspase recruitment domain
cfDNA	Circulating free DNA
PM	Plasma membrane
PAMP	Pathogen-associated molecular pattern
DAMP	Danger-associated molecular pattern
PRR	Pattern-recognition receptors
NLR	Nucleotide-binding leucine-rich repeat
sNLR	Sensor NLR
hNLR	Helper NLR
NOD	Nucleotide oligomerization domain
TNL	Toll Interleukin 1 receptor-NLR
CNL	Coiled-coil-NLR
RNL	Resistance to powdery mildew 8 NLR
LRR	Leucine-rich repeat
NAD+	Nicotinamide adenine dinucleotide
NLRP	NLR family pyrin domain containing 3
PYD	Pyrin domain
HMGB1	High mobility group box 1
IL	Interleukins
GSDMs	Gasdermins
HR	Hypersenstive response
CARD	Caspase recruitment domain
RIPK3	Receptor-interacting protein kinase 3
ROS	Reactive oxygen species
MLKL	Mixed-lineage kinase domain-like
EDS1	Enhanced disease susceptibility 1

**Fig 1:**
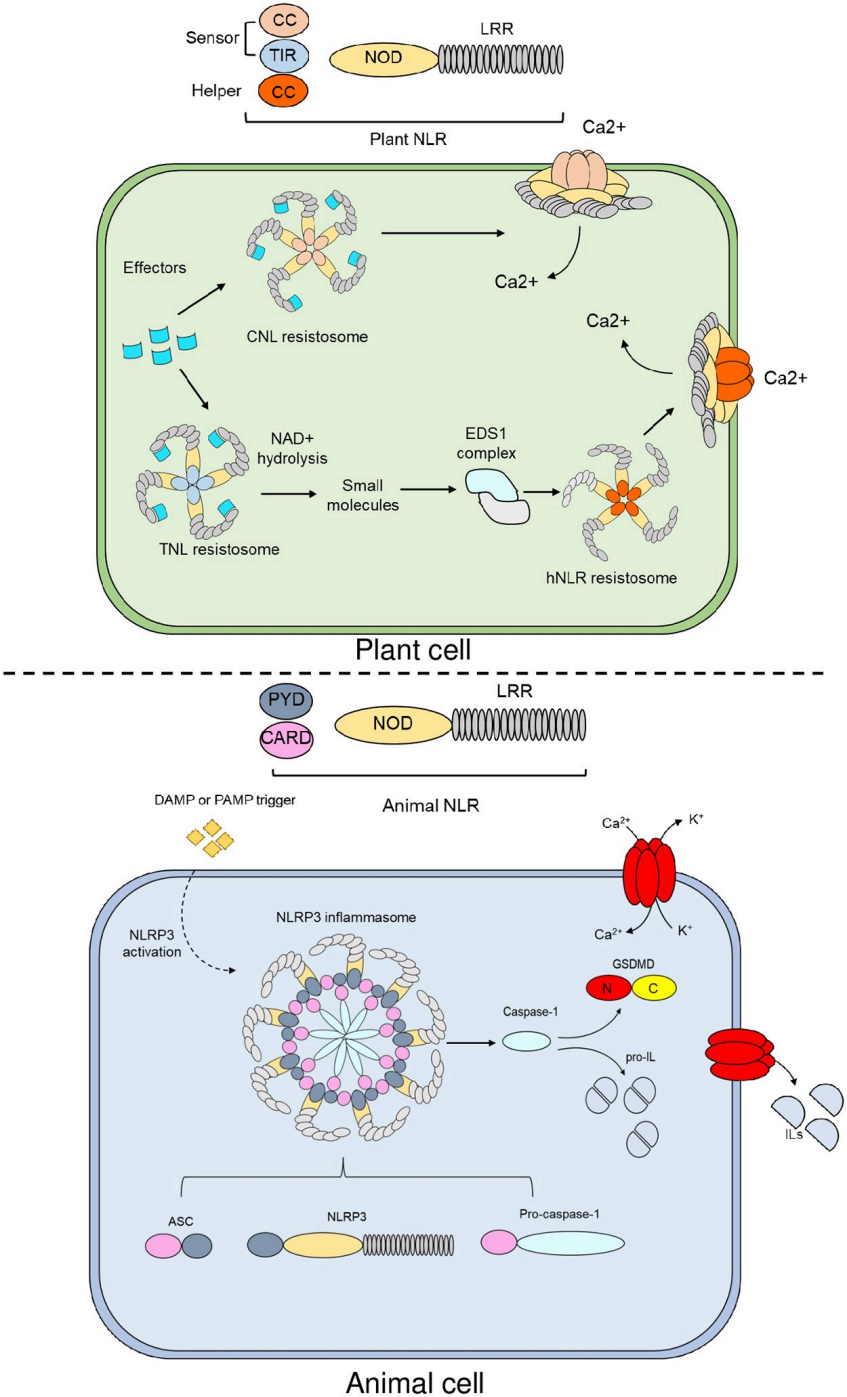
Domain architecture of NLR immune receptors and general activation mechanisms of resistosomes and inflammasomes in plants and animals, respectively. NLRs are modular tripartite immune receptors comprised of an N-terminal signaling domain, a NOD and LRR domain. In plants, NLRs are broadly classified into sNLRs and hNLRs based on their function during the immune response. Sensors are divided into CC- or TIR-NLR, whereas helpers carry a CC domain at their N-terminus. Upon pathogen perception, CNLs oligomerize into a pentameric wheel-like structure, whereas TNLs oligomerize into a tetrameric structure collectively known as resistosomes. While CNLs can sense pathogen effectors and execute cell death by acting as permeable Ca^2+^ channels with no need of hNLRs, TIR domains from TNLs act as NAD+ hydrolases generating by-products or small molecules that bind to EDS1 complexes. Allosteric changes in EDS1 complexes allow interaction with hNLRs. Oligomerization of certain hNLRs into a pentameric resistosome with Ca^2+^channel activity at the PM drive ion flux imbalances that resultin HR-cell death [9,10]. CNL, coiled-coil-NLR; hNLR, helper NLR; HR, hypersensitive response; LRR, leucine-rich repeat; NLR, nucleotide-binding leucine-rich repeat; NOD, nucleotide oligomerization domain; PM, plasma membrane; sNLR, sensor NL; TNL, Toll Interleukin 1 receptor-NLR.

In plants, NLRs are categorized based on their domain composition at the N-terminus and their function during the immune response. NLRs carrying a coiled-coil (CNLs) or a Toll/Interleukin 1-receptor (TIR)-type domain (TNLs) can act as sensor (sNLRs) by perceiving effectors, whereas a subset of CNLs function as helper (hNLRs) by amplifying the downstream immune signal emanating from sensor NLRs or PRRs [4–7]. In animal NLRs, N-terminal domains belong to the death-fold superfamily and mainly include Pyrin and CARD domains [8] (Fig 1).

In animals, the N-terminal domain of NLRs generally harbors either a CARD or a PYRIN domain. Upon recognition of DAMPs or PAMPs, animal NLRs nucleate into heteromeric inflammasome complexes. For instance, the pyrin-containing NLRP3 inflammasome is comprised of a sensor NLR (NLRP3), the adaptor protein Apoptosis-associated speck-like protein containing a caspase recruitment domain (ASC1) and caspase-1. Oligomerization of NLRP3 through homotypic interactions at the NOD recruits the ASC through a PYD-PYD interactions. Conformational changes in ASC allow recruitment of caspase-1 through CARD-CARD interactions, enabling caspase-1 activation. Proteolytically active caspase-1 subsequently cleaves gasdermin D (GSDMD) and pro-ILs that are released into the extracellular space. Insertion of the N-terminal pore-forming domain of GSDMD into the PM leads to nonselective ion fluxes that ultimately results in cellular demise.

## 2. NLRs are activated by oligomerization

NLR activation in both plants and animals involves oligomerization through their N-terminal domains. In mammals, PAMP or DAMP-triggered NLR oligomerization leads to the assembly of the so called “inflammasomes.” These supramolecular structures are comprised of a varying number of NLR molecules depending on the nature of molecule trigger and provide a platform for recruitment and activation of caspases either directly or indirectly through the adaptor protein ASC [11]. Caspase-dependent processing of pro-interleukins (ILs) and GSDMs ultimately results in pyroptosis (Fig 1) (described in section 3).

Upon pathogen effector perception, plant NLRs also assemble into multimeric protein complexes termed “resistosomes” [12–15]. In the case of CNLs, pentameric oligomerization leads to resistosome activation and a concomitant structural switch that results in a funnel-shaped structure that acts as a PM localized cation-selective channel permeable to Ca^2+^ [12,13,16,17]. Altered ion fluxes may act as an important determinant of pathogen-triggered cell death. This indicates that while certain plant immune receptors (sensor CNLs) can act as both sensors and executors of cell death, most animal NLRs require accessory molecules to drive cell death [18] (Fig 1).

Plant TNLs oligomerize into tetrameric protein complexes exhibiting NADase activity (nicotinamide adenine dinucleotide hydrolases) in their TIR domains [19]. By-products or “info-chemicals” derived from TNL-mediated hydrolysis of the metabolic co-factor NAD^+^ can directly bind to heterodimers formed by plant lipase-like proteins with ENHANCED DISEASE SUSEPTIBILITY 1 (EDS1), promoting interactions with helper NLRs [9,10]. Certain helper NLRs can oligomerize into a pentameric resistosome capable of forming pores at the PM and driving ion flux imbalances in a similar way to sensor CNLs [20–22] (Fig 1).

While activated plant resistosomes/NLRs in plants are executors of cell death and localize at the PM membrane (CNLs and hNLRs) where they exert its pore-forming activities, activated animal NLRs (NLRP3 inflammasome) remain cytoplasmic acting as molecular scaffolds for recruitment and activation of accessory molecules that ultimate mediate plasma membrane disruption (Fig 1).

## 3. Immunogenic cell death exists in different flavors

**Fig 2:**
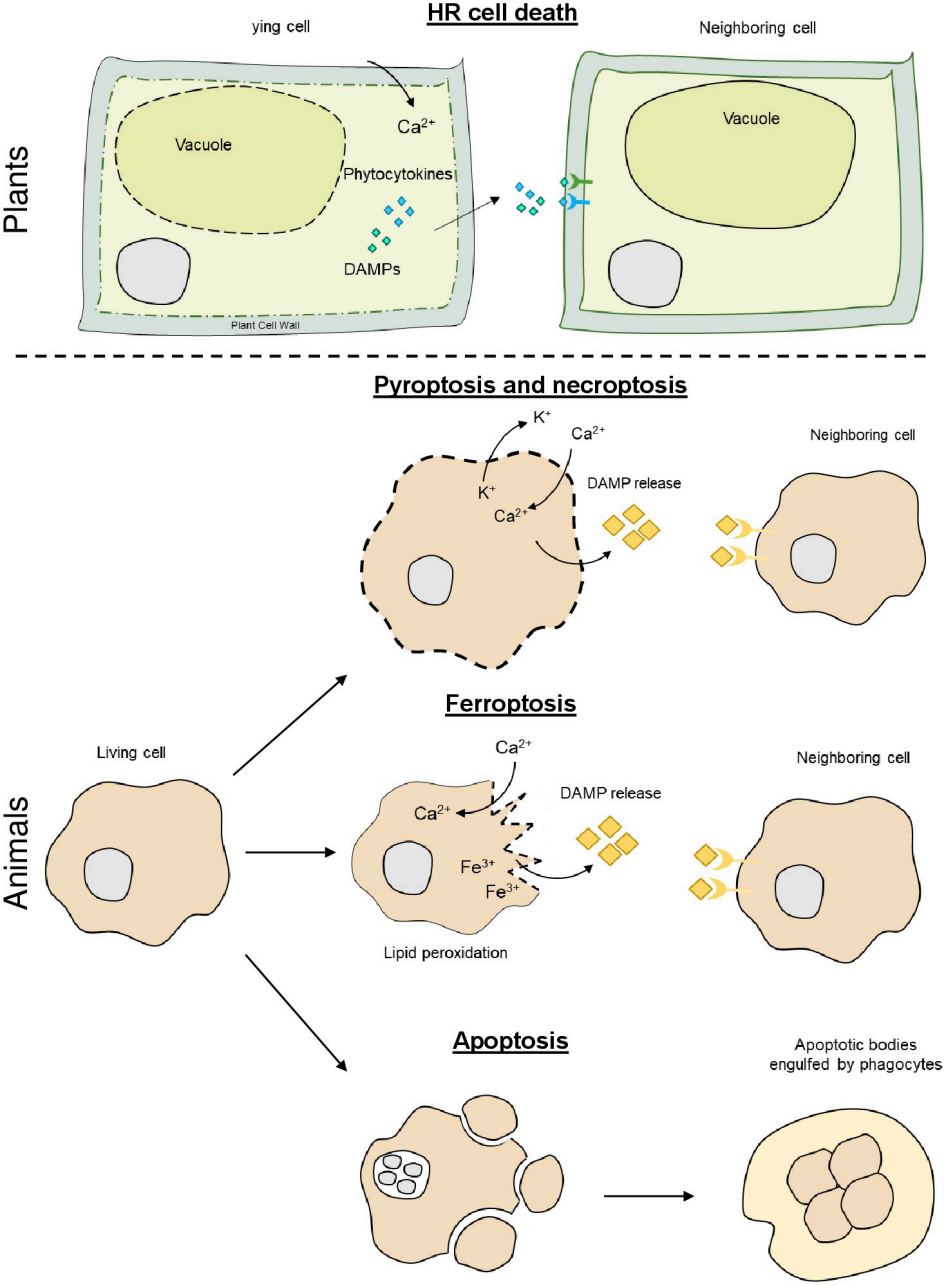
Overview of cell death types and their general features in plants and animals. During HR cell death in plants, ROS accumulation and calcium channel activity exerted by plant resistosomes drive Ca^2+^ entry into the cytoplasm. How intracellular Ca^2+^ spikes lead to downstream cell death features such as loss of chloroplast and mitochondrial and eventually cellular demise is currently unknown. DAMPs and phytocytokines are released from infected/damaged cells and activate defense responses in neighboring cells via perception by surface receptors. Although differentially regulated at the molecular level, pyroptosis and necroptosis are both pro-inflammatory forms of cell death that involve release of cellular content to the extracellular space (DAMP release and inflammatory cytokines). In both cell death modalities, rupture of the plasma allows for the influx and efflux of ions altering homeostasis in the cell. Ferroptosis is an iron-dependent mode of cell death in which peroxidation of lipids cause plasma membrane damage with partial rupture allowing entry of Ca^2+^ ions and release of DAMPs to the extracellular space. Apoptosis is a non-inflammatory and silent form of cell death in which membrane integrity is maintained during cellular dismantling. Cell shrinkage, chromatin condensation, and DNA fragmentation are typical hallmarks of apoptosis. Importantly, plasma membrane blebbing leads to apoptotic bodies that are eventually engulfed and eliminated by phagocytes. DAMP, danger-associated molecular pattern; HR, hypersensitive response; ROS, reactive oxygen species.

In plants, the term hypersensitive response (HR) is used to define a local, pathogen-triggered type of cell death mediated by NLR activation. HR restricts pathogen growth, and hence, it is an important component of plant immunity [23,24]. Broadly, HR involves production of reactive oxygen species (ROS), nitric oxide, and an increase of intracellular calcium, likely mediated by formation of PM pores by resistosomes [12–15,17] (Fig 2). Still, how NLR activation and calcium influxes connects to downstream cell death programs as well as the role of proteolytic enzymes and organelles such as the chloroplast, mitochondria, and the vacuole in this process remains largely unknown.

In animals, pyroptosis, necroptosis, and ferroptosis, unlike apoptosis, are pro-inflammatory cell death programs that involve release of lytic content to the extracellular space and rupture of the plasma membrane prior to cellular demise (Fig 2). Besides their morphological resemblance, their triggers and biochemical executors of the cell death pathways differ [25].

Pyroptosis is activated upon detection of PAMPs or DAMPs by inflammasomes. These multi-protein complexes act as platforms for the activation of caspases that cleave GSDM unleashing its pore-forming domain to form an oligomeric pore at the PM [11]. Pore formation through GSDMD results in cell size increase and subsequent burst, releasing intracellular proteins to the extracellular space.

Necroptosis involves ligand-mediated activation of RECEPTOR-INTERACTING PROTEIN KINASE 3 (RIPK3) that phosphorylates the pseudo-kinase MIXED LINEAGE KINASE DOMAIN-LIKE (MLKL) [26]. Phosphorylation drives interaction of MLKL with the PM where it oligomerizes and forms a necroptotic pore [26]. Pore formation also results in the release of intracellular content, including pro-inflammatory ILs, eventually leading to cellular demise. Interestingly, plants possess a conserved protein family resembling animal MLKLs that participate in immunity, indicating a potentially common mode of action with animal MLKLs [27].

Ferroptosis is a lytic, pro-inflammatory cell death that involves iron-dependent peroxidation of lipids associated with loss of PM integrity and ion influxes [28] (Fig 2). In plants, a ferroptosis-like process has been reported in response to NLR-mediated recognition of a fungal pathogen [29]. Conservation between plant and animal ferroptosis may unfold as the mechanisms and players of the process become fully elucidated.

Apoptosis is an immunologically silent form of cell death in which gradual dismantling of the cell content leads to morphological features such as cytoplasmic shrinkage, chromatin condensation, and DNA fragmentation [30]. As opposed to other cell death programs, PM integrity is retained throughout the cell death process. Eventually, membrane blebbing results in cell fragmentation giving rise to “apoptotic bodies” that are engulfed and eliminated by phagocytes (Fig 2). Apoptosis initiation culminates in activation of effector caspases and concomitant cell death [25]. Inhibition of caspases is an important target for pathogens to prevent apoptosis and maintain their replicative niche. It is thus not surprising that caspases have evolved as versatile molecular switches that can resort to pro-inflammatory cell death when apoptosis is blocked. In fact, an increasing number of immunogenic cell death modalities, deeply interlinked between them, is emerging as a central determinant of tissular/systemic responses [31].

## 4. Loss of plasma membrane/endomembrane integrity is a key step of immunogenic cell death

Loss of plasma membrane/endomembrane integrity is a common hallmark between plant and animal immunogenic cell death. In animals, pore formation at the PM constitutes an execution step of pro-inflammatory cell death and it involves GSDMD and MLKL in pyroptosis and necroptosis, respectively. During pyroptosis, the N-terminal portion of GSDMD, cleaved by caspase-1, directly inserts into the PM, where it self-associates and forms ring-shaped pores (approximately 20 nm) [32]. These large pores allow the release of pro-inflammatory molecules (cytokines, alarmins) and cause cell lysis. In the case of necroptosis, phosphorylated MLKL interacts with the PM, although the pore structure remains unresolved. Therefore, its oligomeric state in membranes and how it mediates permeabilization remain not fully elucidated. MLKL pores drive calcium and sodium influx and potassium efflux from the cell followed by water influx, resulting in a cell burst typical of necroptosis [33,34]. Ferroptosis also involves loss of integrity and partial rupture of the PM, which has been associated with iron-dependent peroxidation of phospholipids [28].

In plants, it has been demonstrated that CNL pentameric resistosomes can drive membrane pore formation. Oligomerization of CNLs results in a structural switch of the N-terminus of each monomer that then projects out of the resistosome plane. The funnel-shaped structure can insert into membranes forming a small pore (approximately 1 nm) that can act as a cation-selective channel permeable to Ca^2+^ [16,17,20]. Pore formation and subsequent Ca^2+^ influx may activate a cell death program as described for ferroptosis. In sum, current evidence suggests that transient or permanent pore formation at the PM and permeabilization constitutes a common mechanism to execute cell death both in plant and animal cells.

## 5. Dying cells release signaling molecules important for immunity

Immunogenic cell death results in the release of signaling molecules, which activate immunity in surrounding/distal tissues and is therefore an important mechanism to counteract invading agents. In animals, immunogenically dying cells release DAMPs such as nuclear HIGH MOBILITY GROUP BOX 1 PROTEINS (HMGB1), ATP or circulating free DNA (cfDNA), among others. In addition, pyroptotic and necroptotic cells release pro-inflammatory cytokines. DAMP release appears tightly controlled and not a mere consequence of cell lysis as originally considered. In this sense, a growing body of evidence indicates that different types of lytic cell death will release a distinct signature of pro-inflammatory molecules [29,35].

During plant immune responses, a broad range of DAMPs and phytocytokines are released from infected/damaged cells and activate defense responses locally and in surrounding tissues [36,37]. DAMPs include nucleotides, sugars, and amino acids, while phytocytokines comprise endogenous signaling peptides actively generated upon maturation of the propeptide by a protease and subsequently perceived by cell surface receptors. Expression of phytocytokine precursors is in fact up-regulated upon MAMP treatments or pathogen attack, constituting an early immune response [38]. Among phytocytokines, those peptides that do not contain a secretory signal may reach the extracellular space after cell lysis via not yet identified mechanisms. Research in recent years has evidenced that multitude of phytocytokines may in fact regulate immune responses, although very few have been characterized to date, such as some PLANT ELICITOR PEPTIDES (PEPs) or RAPID ALCALINIZATION FACTORS (RALFs) [37].

An exciting avenue for future research is whether specific DAMPs/phytocytokines emanate from dying cells, and if so, how do they communicate with neighboring cells and whether specific signatures exist depending on the particular plant–pathogen interaction. Also, it remains unclear what is the exact effect of phytocytokines in neighboring cells: Do they promote cell death or they are rather acting as pro-survival molecules acting for example in tissue repair? In coming years, we may witness how increasing knowledge on plant HR is translated into disease resistance in the field, in the same way that basic knowledge on pro-inflammatory cell death in animals is leading to novel therapeutics.
